# Spatial and temporal epidemiology of clinical malaria in Cambodia 2004–2013

**DOI:** 10.1186/1475-2875-13-385

**Published:** 2014-09-30

**Authors:** Richard J Maude, Chea Nguon, Po Ly, Tol Bunkea, Pengby Ngor, Sara E Canavati de la Torre, Nicholas J White, Arjen M Dondorp, Nicholas PJ Day, Lisa J White, Char Meng Chuor

**Affiliations:** Mahidol-Oxford Tropical Medicine Research Unit, Faculty of Tropical Medicine, Mahidol University, Bangkok, Thailand; Centre for Tropical Medicine, Nuffield Department of Medicine, University of Oxford, Oxford, UK; National Centre for Parasitology, Entomology and Malaria Control, Phnom Penh, Cambodia; Department of Clinical Tropical Medicine, Faculty of Tropical Medicine, Mahidol University, Bangkok, Thailand

**Keywords:** Cambodia, Malaria, Falciparum, Vivax, Epidemiology, Spatial

## Abstract

**Background:**

Artemisinin-resistant *Plasmodium falciparum* malaria has recently been identified on the Thailand-Cambodia border and more recently in parts of Thailand, Myanmar and Vietnam. There is concern that if this resistance were to spread, it would severely hamper malaria control and elimination efforts worldwide. Efforts are currently underway to intensify malaria control activities and ultimately eliminate malaria from Cambodia. To support these efforts, it is crucial to have a detailed picture of disease burden and its major determinants over time.

**Methods:**

An analysis of spatial and temporal data on clinical malaria in Cambodia collected by the National Centre for Parasitology, Entomology and Malaria Control (CNM) and the Department of Planning and Health Information, Ministry of Health Cambodia from 2004 to 2013 is presented.

**Results:**

There has been a marked decrease of 81% in annual cases due to *P. falciparum* since 2009 coinciding with a rapid scale-up in village malaria workers (VMWs) and insecticide-treated bed nets (ITNs). Concurrently, the number of cases with *Plasmodium vivax* has greatly increased. It is estimated that there were around 112,000 total cases in 2012, 2.8 times greater than the WHO estimate for that year, and 68,000 in 2013 (an annual parasite incidence (API) of 4.6/1000). With the scale-up of VMWs, numbers of patients presenting to government facilities did not fall and it appears likely that those who saw VMWs had previously accessed healthcare in the private sector. Malaria mortality has decreased, particularly in areas with VMWs. There has been a marked decrease in cases in parts of western Cambodia, especially in Pailin and Battambang Provinces. In the northeast, the fall in malaria burden has been more modest, this area having the highest API in 2013.

**Conclusion:**

The clinical burden of falciparum malaria in most areas of Cambodia has greatly decreased from 2009 to 2013, associated with roll-out of ITNs and VMWs. Numbers of cases with *P. vivax* have increased. Possible reasons for these trends are discussed and areas requiring further study are highlighted. Although malaria surveillance data are prone to collection bias and tend to underestimate disease burden, the finding of similar trends in two independent datasets in this study greatly increased the robustness of the findings.

**Electronic supplementary material:**

The online version of this article (doi:10.1186/1475-2875-13-385) contains supplementary material, which is available to authorized users.

## Background

Epidemiological data on malaria over time are essential to planning of malaria control and elimination. In Cambodia, the control and elimination of malaria has garnered much attention due the recent discovery of artemisinin resistance in Pailin Province on the Thai border [1]. This area has historically been the source of resistance to other anti-malarial drugs and if artemisinin resistance were also to spread it would be a major threat to malaria control and elimination efforts worldwide [2, 3]. Resistance has since been identified in elsewhere in Cambodia and parts of Thailand [4], Myanmar [5] and Vietnam [6]. Mathematical modelling has been employed to predict the most effective interventions to try to eliminate artemisinin-resistant malaria parasites [7, 8]. The modelling predictions are heavily dependent on accurate information on baseline malaria epidemiology.

Since 1992, the Department of Planning and Health Information has used a standardized system for collecting monthly data on malaria cases across the whole of Cambodia into a centralized database, the Health Information System (HIS). This includes clinical cases in each province who attend public sector health facilities. Since 2004, the National Centre for Parasitology, Entomology and Malaria Control (CNM) has rolled out a system of Village Malaria Workers (VMWs) across the areas of the country with highest malaria prevalence. These provide diagnosis and treatment at the village level and report their activities through the Malaria Information System (MIS) on cases diagnosed and treated to CNM. Between these two sources, HIS and MIS, a detailed picture of malaria in Cambodia over time can be gleaned.

This study analysed data collected through HIS and MIS from 2004 to 2013 to produce a picture of the spatial and temporal epidemiology of malaria in Cambodia.

## Methods

Data were extracted from the HIS and MIS databases and CNM annual reports on numbers of malaria cases and malaria control activities from January 2004 to December 2013. For clarity in Figures throughout this manuscript, the MIS data are referred to as ‘VMW’. Data on population and average rainfall in Cambodia were also collected [9, 10]. These were analysed using Microsoft Excel 2013 and GraphPad Prism Version 6.04 and maps produced using ARC GIS 10.2.

### Statistics

Statistical analyses were performed using GraphPad Prism Version 6.04. Correlations were examined using Spearman R and significance was set at the 5% level.

## Results

### Health information system

#### Total cases

From 2004–2013, 1,667,188 people tested for malaria in Cambodia were recorded by the HIS system. Of these, 496,070 (29.8%) were positive, 385,045 (77.6%) with *Plasmodium falciparum* and 139,254 (28.1%) with *Plasmodium vivax*. 28,229 (5.7%) had mixed infection. 666,019 people were recorded as treated for malaria. Thus at least 169,949/666,019 (25.5%) of those treated for malaria did not have parasitological confirmation of malaria. During this period, there were 2085 (0.5%) recorded deaths from malaria. The yearly average number of confirmed malaria cases in the HIS was 49,607. Diagnosis was performed using microscopy or a rapid diagnostic test (RDT). The proportion of HIS recorded patients diagnosed by microscopy fell from 52% in 2007 to 14% in 2013.

#### Seasonality

**Figure 1:**
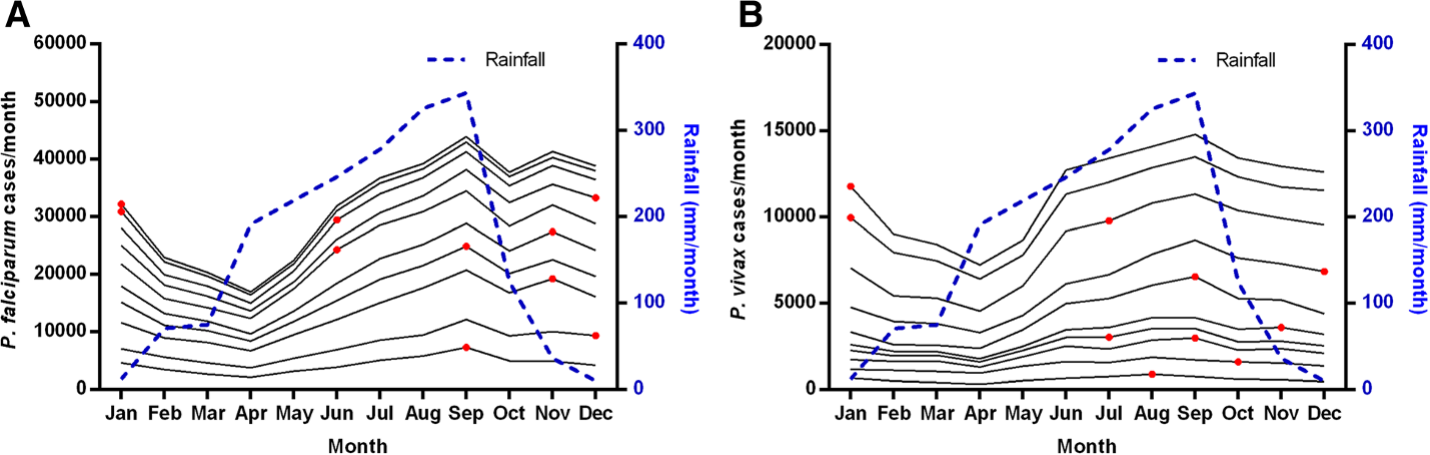
**Cumulative seasonal pattern of malaria cases. A** *P. falciparum* and **B** *P. vivax* malaria cases from HIS and average monthly rainfall in Cambodia from 2004 (bottom) to 2013 (top). Each band represents a single year from 2004 (bottom) to 2013 (top). The month with the highest number of cases in each year is indicated by a red dot.

The numbers of cases of both falciparum and vivax malaria were highly seasonal with a broad peak and the highest monthly numbers from June to January, the time of highest rainfall (Figure 1). In most years, the peaks in cases with falciparum and vivax malaria were in different months. The month with the highest number of cases varied from June to January with two peaks for both species in some years.

#### Temporal trends

**Figure 2:**
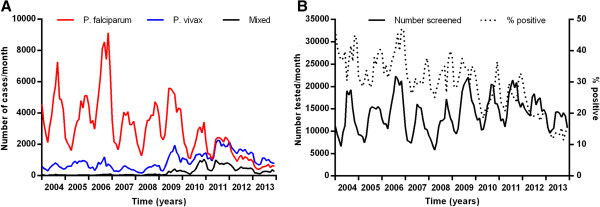
**Reported malaria cases and people tested. A** Monthly numbers of reported malaria cases and **B** people tested for malaria in Cambodia HIS from 2004–2013.

Overall from 2004 to 2013, there has been a marked decrease in *P. falciparum* and an increase in *P. vivax* (Figure 2).

Much of this decrease was from 2011–2013 during which the total number of cases with either species fell by 59% (Figure 2A). For *P. falciparum*, from 2004 to 2009 there was no clear overall trend in the annual number of cases. Since 2009, there has been a marked and steady decrease in annual cases of 81% (p = 0.006). The number of cases with *P. vivax* remained stable from 2004 to 2008. It then increased from 2008 to 2011 (p = 0.01), the total number being 4.9 times greater in 2011 than in 2008. This was followed by a 50% decline in *P. vivax* cases from 2011 to 2013. The proportion of cases due to *P. vivax* was 12% in 2004 and this remained steady until it began to rise from 23% in 2009 to 67% in 2013.From 2004 to 2011 the number of people tested and proportion of tests positive for malaria did not change (Figure 2B, p = 0.11), indicating that the overall trends in cases detected were not an artefact due to changing surveillance efforts. Since 2011, the proportion of tests which were positive has steadily fallen, suggesting a relative intensification of malaria testing and consistent with a true decline in incidence of clinical malaria.

**Figure 3:**
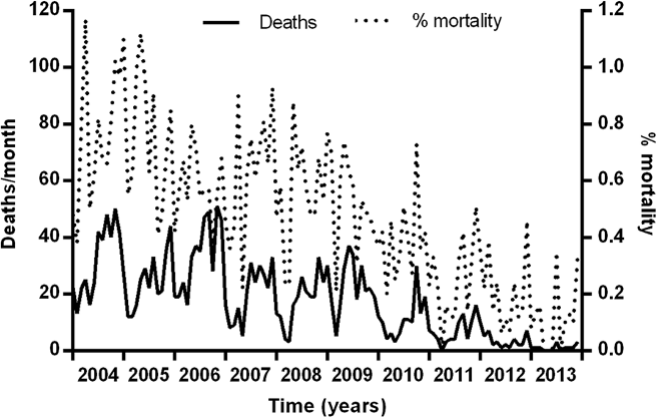
**Numbers of deaths and percent mortality from** ***P. falciparum*** **malaria in Cambodia from 2004 to 2013.**

From 2004 to 2013, the number of deaths from malaria and % mortality from *P. falciparum* decreased, p = 0.0003 and p < 0.0001, respectively (Figure 3). As the parasite species in fatal cases was not recorded, it was assumed all deaths were due to *P. falciparum*.

### Spatial distribution

**Figure 4:**
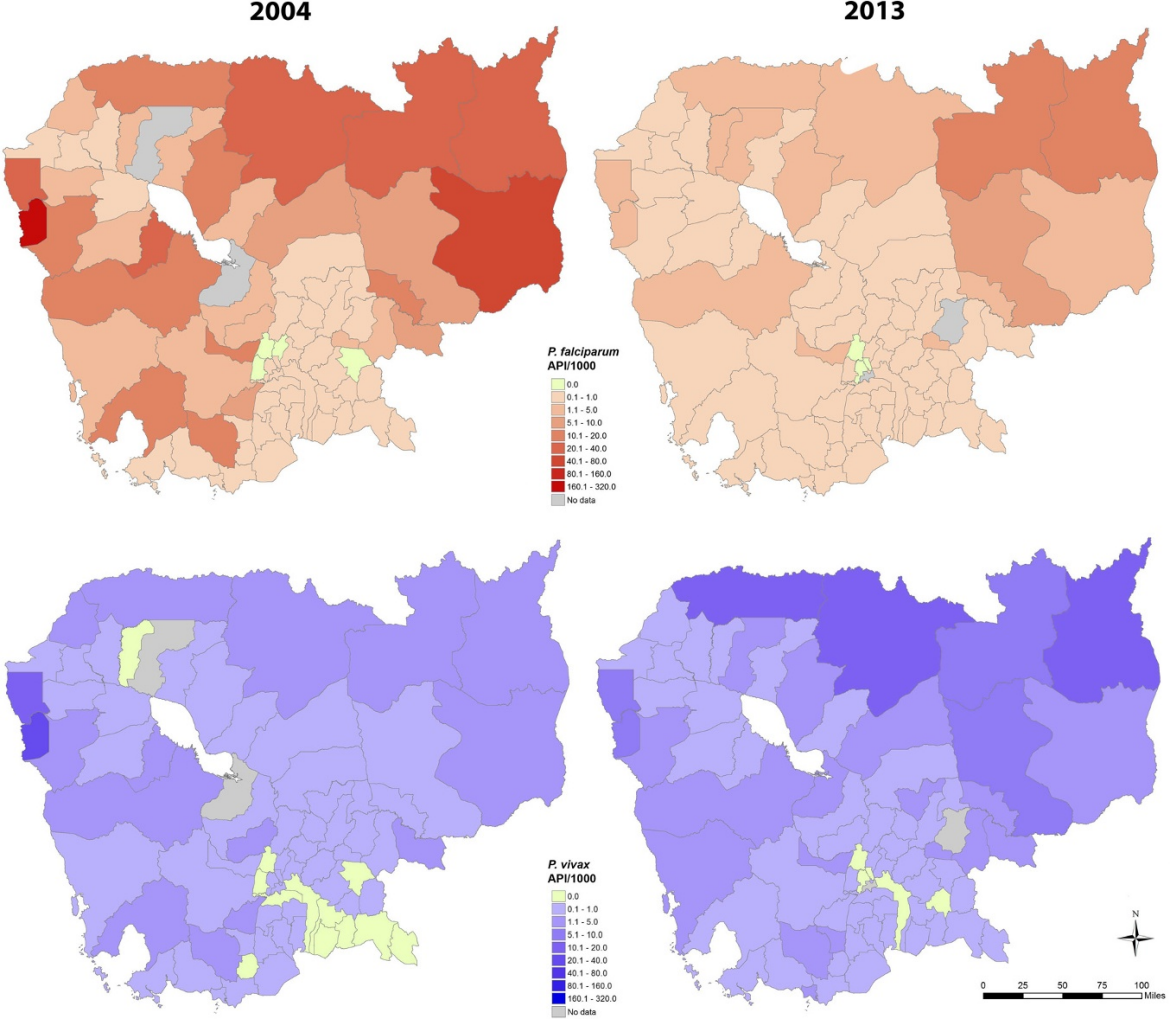
**Geographical distribution of** ***P. falciparum*** **and** ***P. vivax*** **malaria in Cambodia in 2004 and 2013, shown as API per 1000 population.**

**Figure 5:**
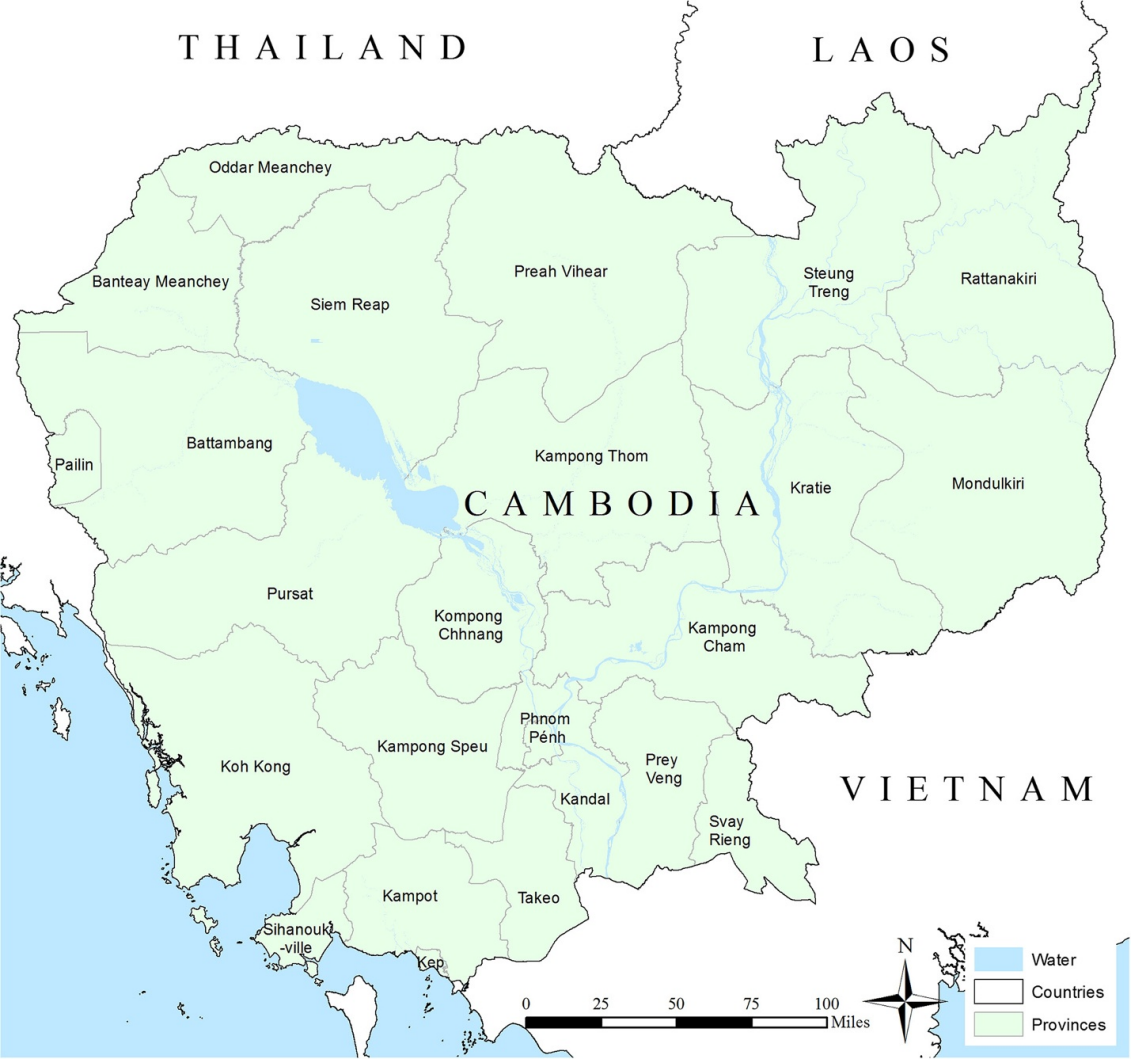
**Reference map of Cambodia showing provinces and neighbouring countries.**

Almost all ODs had cases of malaria in 2004 and 2013 (Figures 4, and 5 for reference). 3/75 ODs had no cases of malaria in 2004 and 3/77 in 2013. The spatial distribution of malaria cases was similar for *P. falciparum* and *P. vivax*. In 2013, the highest transmission intensity for both species was in the north and northeast of the country, some areas of which remain heavily forested. The four operational districts (ODs) with the highest transmission for *P. falciparum* in descending order were Rattanakiri (annual parasite incidence (API) 13.6/1000), Steung Treng (API 10.2/1000), Kratie (API 9.1/1000) and Preah Vihear (API 4.4/1000). The four ODs with the highest transmission for *P. vivax* in 2013 were Preah Vihear (API 17.1/1000), Rattanakiri (API 11.3/1000), Samrong (API 10.1/1000) and Steung Treng (API 8.8/1000). Although the four provinces in the northeast of Cambodia (Mondulkiri, Rattanakiri, Steung Treng and Kratie) had only 3% of the population, they had 43% of the falciparum malaria and 25% of the vivax malaria cases in 2013

The geographical distribution of malaria in Cambodia has changed from 2004 to 2013 (Videos in Additional files 1 and 2).

**Figure 6:**
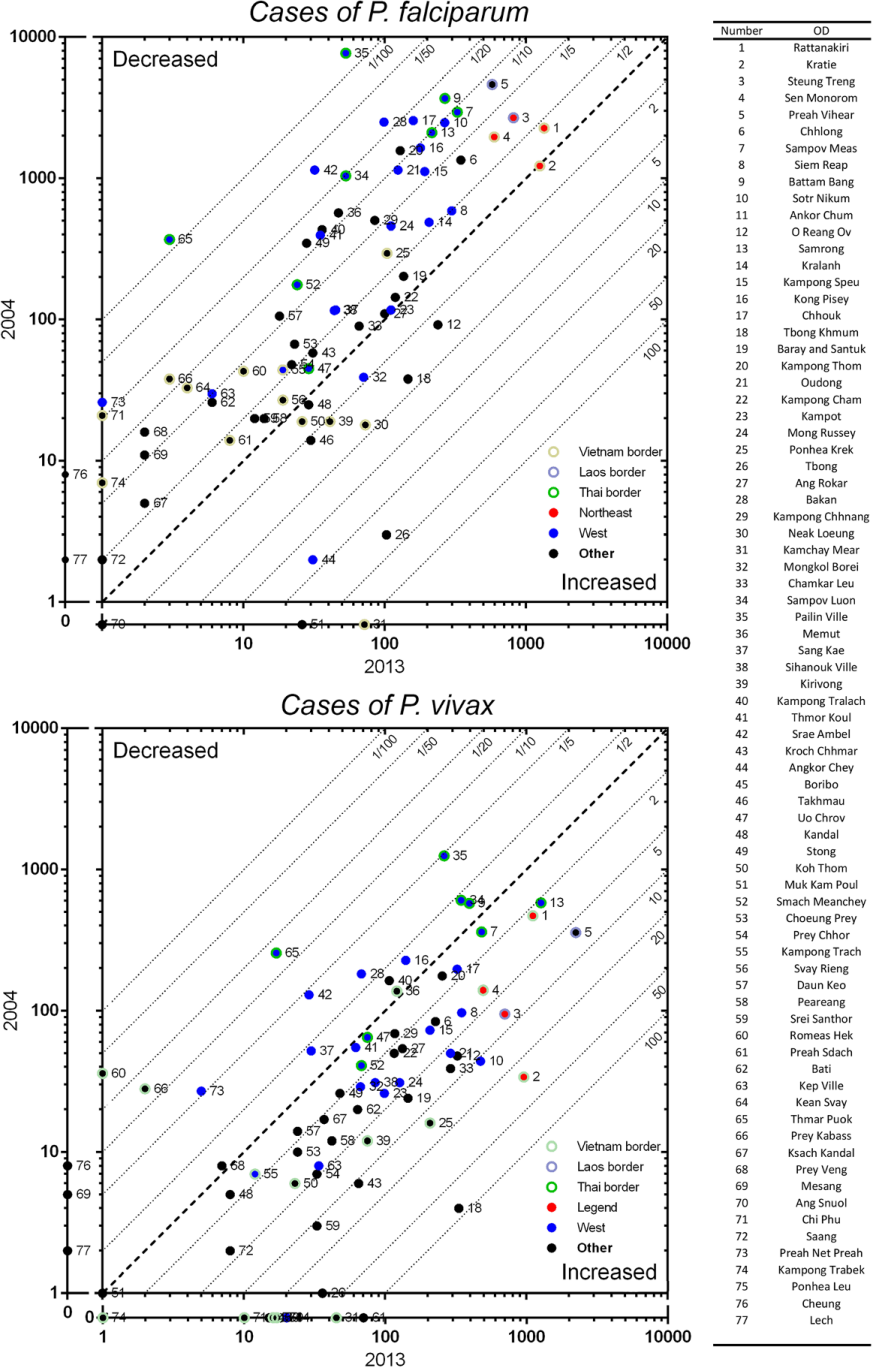
**Numbers of cases of** ***P. falciparum*** **and** ***P. vivax*** **in each OD in 2004 compared to 2013.** The bold diagonal line indicates no change from 2004–2013, points above it represent a decrease in cases from 2004 to 2013 and points below it an increase. It can be seen that in most ODs, *P. falciparum* has decreased and *P. vivax* increased.

There were several major trends. In most ODs, *P. falciparum* decreased from 2004 to 2013 and *P. vivax* increased, although there were some exceptions. From 2004 to 2013 *P. falciparum* remained widespread across Cambodia with low and unstable transmission in the southeast. *P. vivax* transmission was much less stable, with low and unstable transmission in many areas, particularly in a band from the southeast to the northwest of the country.The changes in numbers of cases from 2004 to 2013 for each OD are presented in Figure 6.

**Figure 7:**
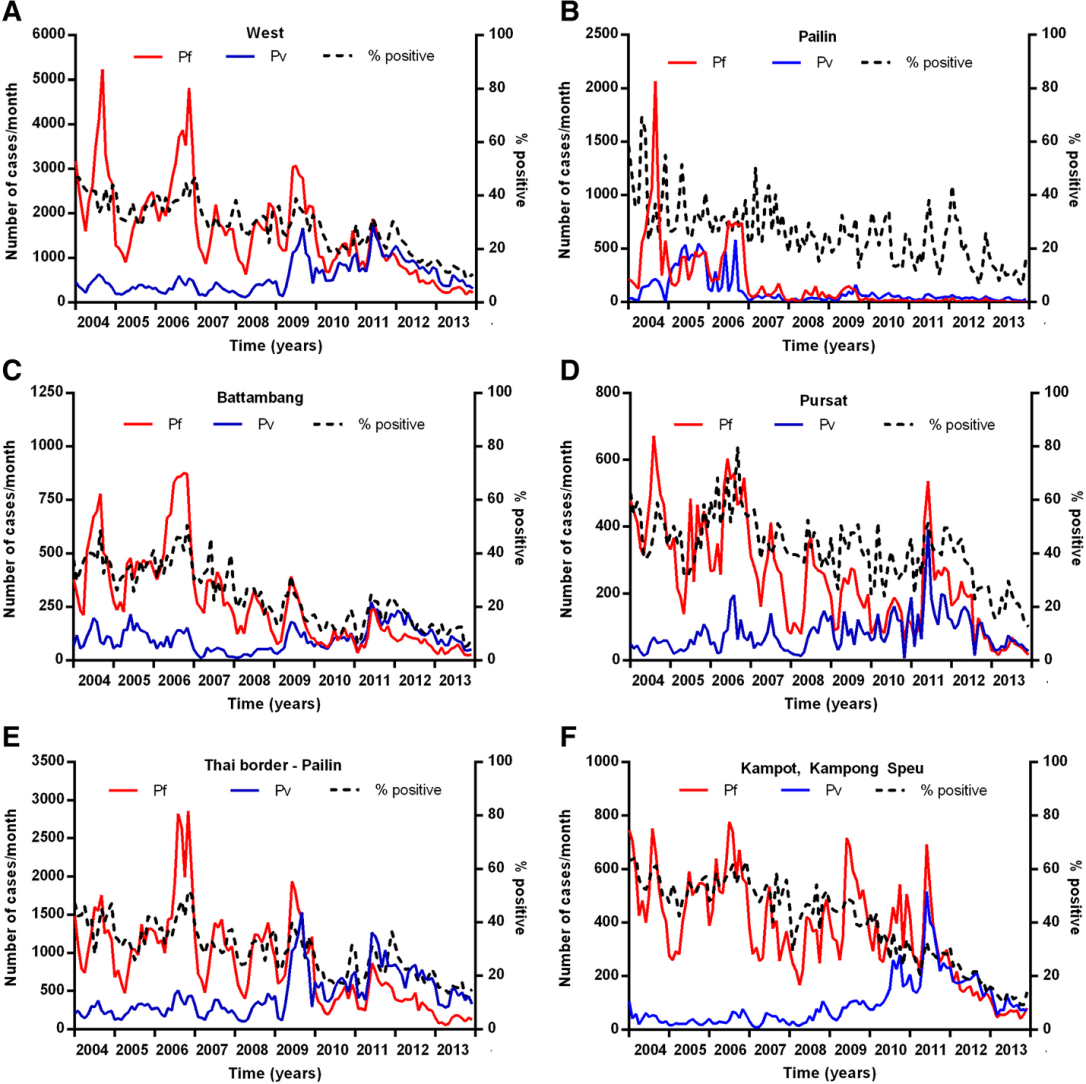
**Number of cases of** ***P. falciparum*** **and** ***P. vivax*** **malaria and proportion of tests positive for malaria in selected regions of western Cambodia. A** west, **B** Pailin Province, **C** Battambang Province, **D** Pursat Province, **E** the Thai-Cambodian border (excluding Pailin) and **F** Kampot & Kampong Speu Provinces.

**Figure 8:**
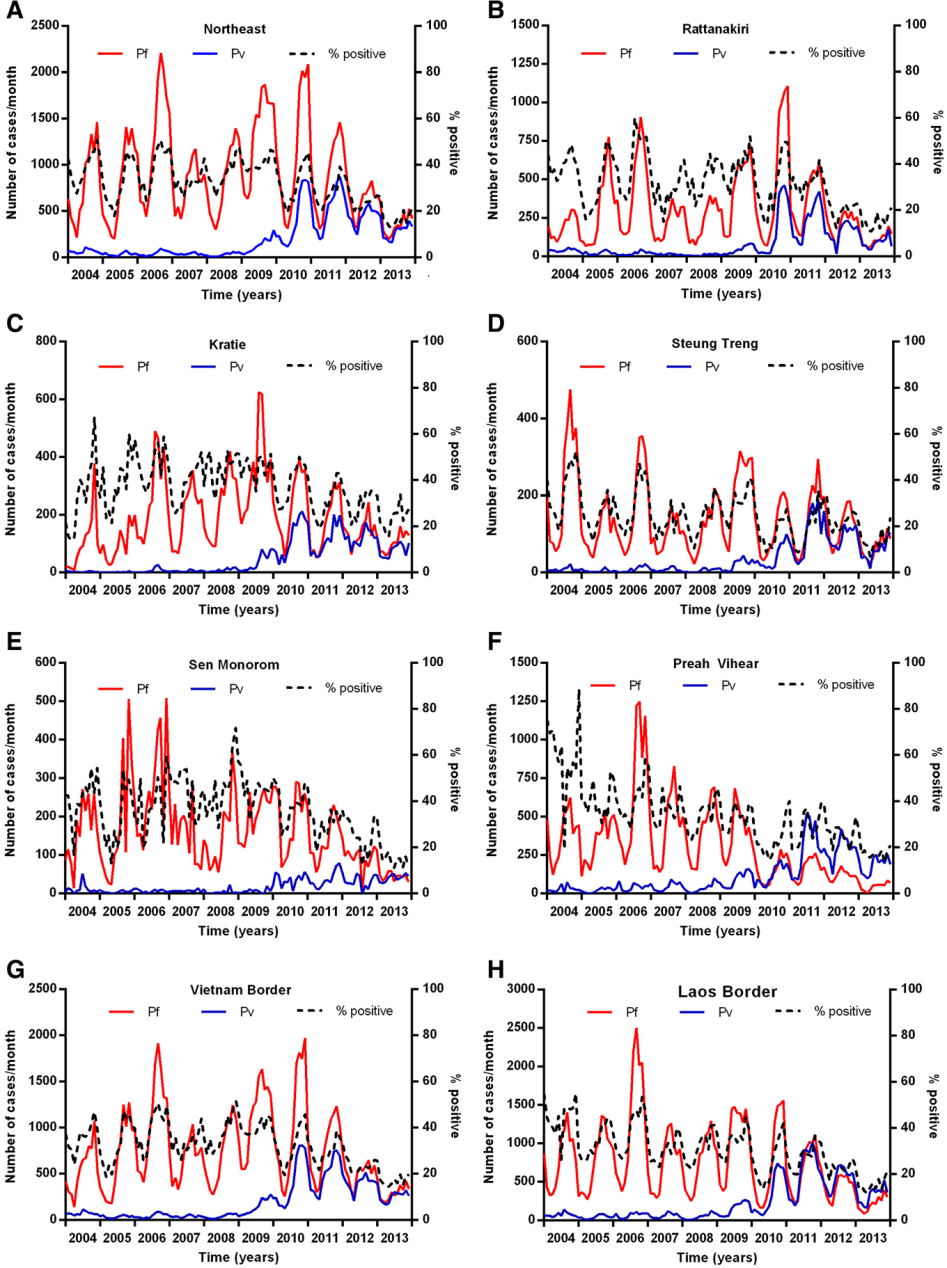
**Number of cases of** ***P. falciparum*** **and** ***P. vivax*** **malaria and proportion of tests positive for malaria in selected regions of northern and northeastern Cambodia. A** northeast Cambodia, **B** Rattanakiri Province, **C** Kratie Province, **D** Steung Treng Province, **E** Sen Monorom Province, **F** Preah Vihear OD, **G** the Vietnam-Cambodian border and **H** the Laos-Cambodia border.

In the west of Cambodia (Pailin, Battambang, Banteay Meanchey, Pursat, Siem Reap and Oddar Meanchey provinces) there was a marked and steady decrease in *P. falciparum* from 2007 onwards (Figure 7A). This decrease was most marked in Pailin (Figure 7B), the OD with the highest transmission intensity from 2004–2006, and to a lesser degree in the surrounding province of Battambang (Figure 7C) with historically much lower transmission than Pailin. Also in Pailin, there was no increase in cases of *P. vivax* from 2008 to 2011, unlike most other malaria endemic areas of the country. In parts of the west of Cambodia, the decrease in *P. falciparum* was less marked, for example Pursat Province (Figure 7D). The ODs along the border with Thailand (in the North and West), with the exception of Battambang and Pailin, did not experience a sustained decrease in transmission until 2012 (Figure 7E). This was also the case in the provinces of Kampot and Kampong Speu in the South (Figure 7F). There was an increase in *P. falciparum* malaria in the Northeast of Cambodia from 2007–2010 (Figure 8A, Rattanakiri from 2007–2010 (Figure 8B) and Kratie (Figure 8C) and Steung Treng (Figure 8D) from 2007–2009 with no increase in Sen Monorom (Figure 8E). Although cases in the Northeast have since decreased by 58%, it remains the area with the highest transmission. The seasonality in monthly numbers of cases was also much more prominent in the North and East than in the West. In Preah Vihear Province in the North (Figure 8F), the relative increase in cases from 2008–2011 was particularly marked. *P. vivax* transmission was most stable in the ODs along the borders with Thailand (Figure 7E), Vietnam (Figure 8G) and Laos (Figure 8H).

### Malaria control activities

**Figure 9:**
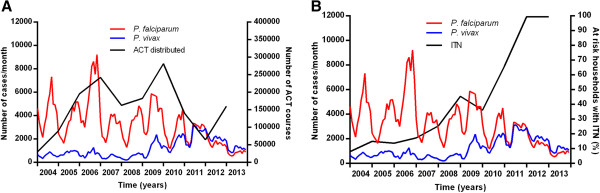
**Malaria control activities and monthly numbers of cases in Cambodia. A** ACT courses distributed per year. **B** proportion of households in high risk areas with ITNs.

During the period of the data collection, the sale of artemisinin combination therapy (ACT) for treatment of malaria increased, insecticide-treated bed net distribution was scaled up and long-lasting insecticide treated bed nets were introduced in 2007 (Figure 9). Detailed data on actual numbers of ACT taken and bed net usage were not included in this study, and the figures for sales and distribution were used as an approximate surrogate. There were no clear relationships between the number of courses of ACT distributed and changes in the number of malaria cases reported through HIS.

### Village malaria workers

**Figure 10:**
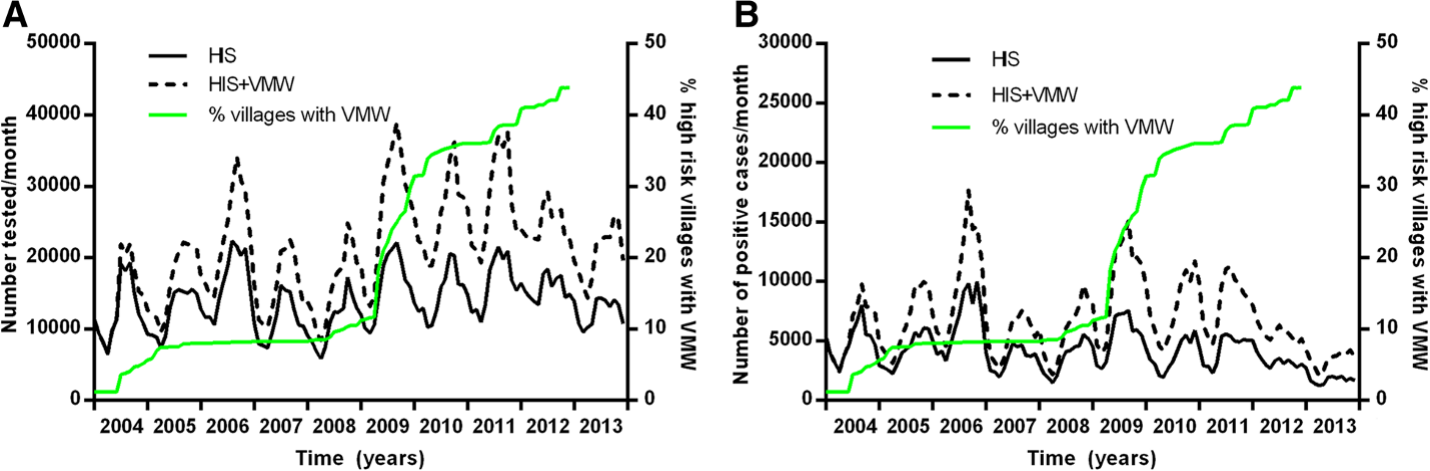
**Number tested for malaria over time in HIS and in VMW data. A** Individuals tested for malaria and **B** positive cases over time in HIS (solid lines) and HIS plus VMW data (dotted line) with % of villages in Cambodia with a VMW (green line).

**Figure 11:**
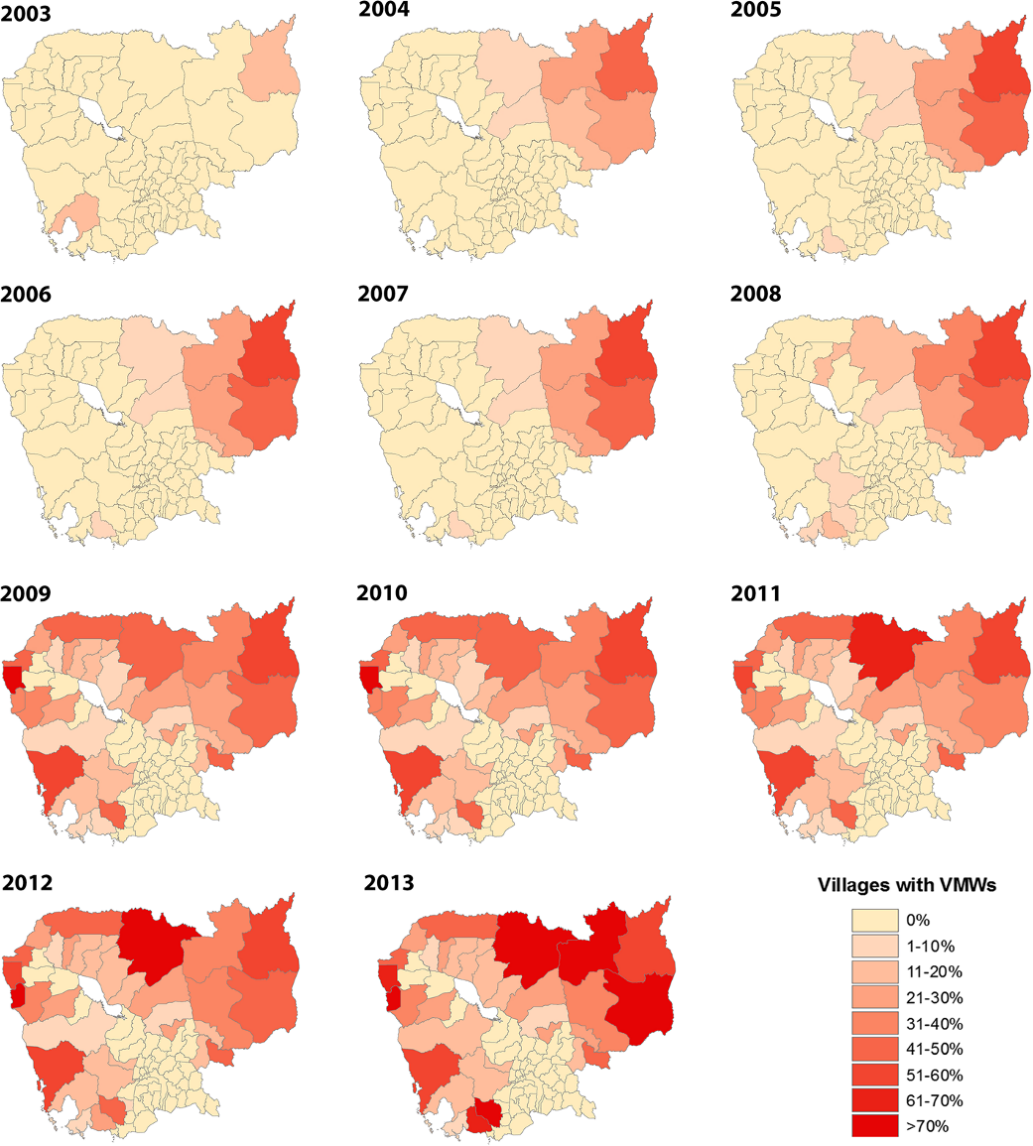
**Coverage with VMWs by OD in Cambodia from 2003–2013.**

The number of VMWs has gradually increased in Cambodia since their official introduction in 2004 (Figure 10), following small-scale pilots from 2001–2003. Being predominantly in more remote areas (≥5 kilometres away from a health centre or one hour walking time) with higher malaria prevalence, they covered 2030 villages by the end of 2013 (14% of the national total and 32% of the villages in the 34 ODs currently included in the VMW programme, Figure 11). In 2013 49% of total cases were detected by VMWs. Since their introduction, VMWs have detected 331,319 RDT positive cases of malaria. From 2004–2013, 23,055 (7.0%) of those detected were referred to government facilities due to having severe malaria or having another clinical episode of malaria within one month of initial treatment. Most of those referred would thus also have been captured in the HIS data. Thus at least 308,264 additional cases of malaria not included in the HIS data were detected by VMWs from 2004 to 2013. This gives a total of 802,540 cases nationwide from 2004 to 2013. With the same rate of referral there would have been 40,278 total cases with an API of 2.7 in 2013.

**Figure 12:**
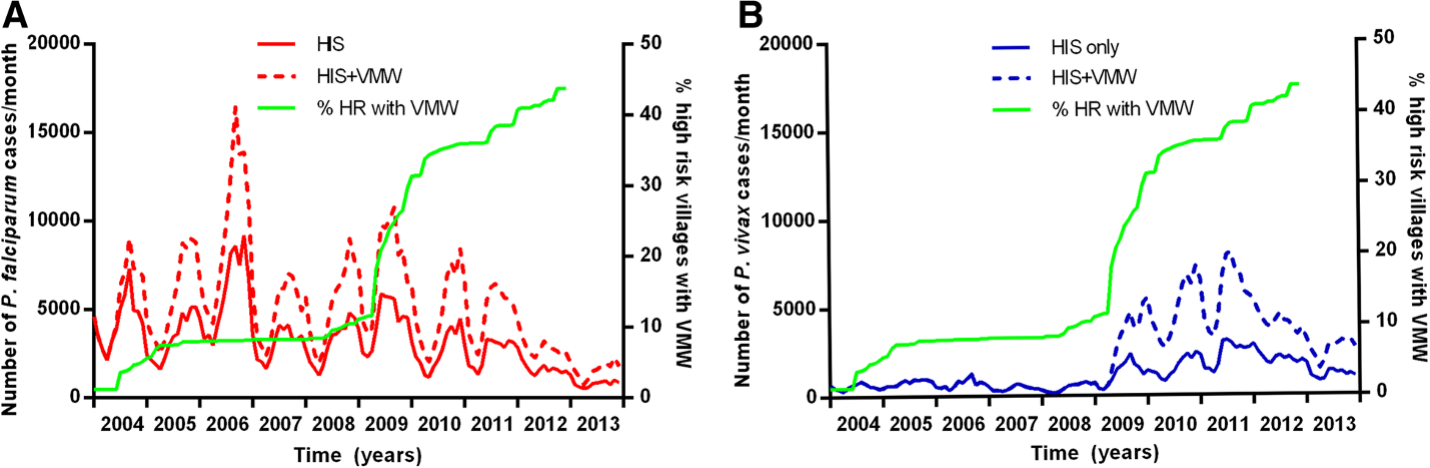
**Additional monthly malaria cases detected by VMWs (MIS) in Cambodia by species. A** *P. falciparum* and **B** *P. vivax*.

Until 2009, VMWs used only parasite histidine-rich protein 2 (p-HRP2) based *P. falciparum* RDTs (Paracheck Pf®) to test people for malaria thus only *P. falciparum* cases were detected (Figure 12). Since 2009, these have gradually been replaced by lactate dehydrogenase (LDH) plus HRP2-based RDTs (CareStart™), which can detect both *P. falciparum* and *P. vivax*.

**Figure 13:**
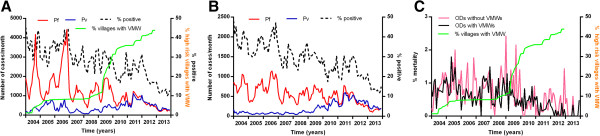
**Malaria cases and deaths over time in villages with and without VMWs.** Cases reported through HIS in ODs **A** with and **B** without VMWs. **C** % mortality in ODs with and without VMWs.

With the scale-up of VMWs, the number of *P. falciparum* and *P. vivax* malaria cases detected by HIS in ODs with VMWs has markedly decreased since 2006 (Figure 13A) compared to those without VMWs (Figure 13B). However, the number of individuals recorded as tested for malaria in the HIS system in these ODs remained steady and the number of cases detected by VMWs fell from 2009. This suggests that, rather than being due to infected individuals attending VMWs instead of government clinics and hospitals, the fall in cases in the HIS system was due to a true decrease in malaria. Numbers of cases of *P. vivax* malaria increased in both from 2009–2011 and numbers of cases of both species have decreased similarly in all areas since 2012. The decrease in malaria mortality was more marked in ODs with VMWs since the large scale-up of coverage with VMWs from 2008 – 2009 (Figure 13C).

**Figure 14:**
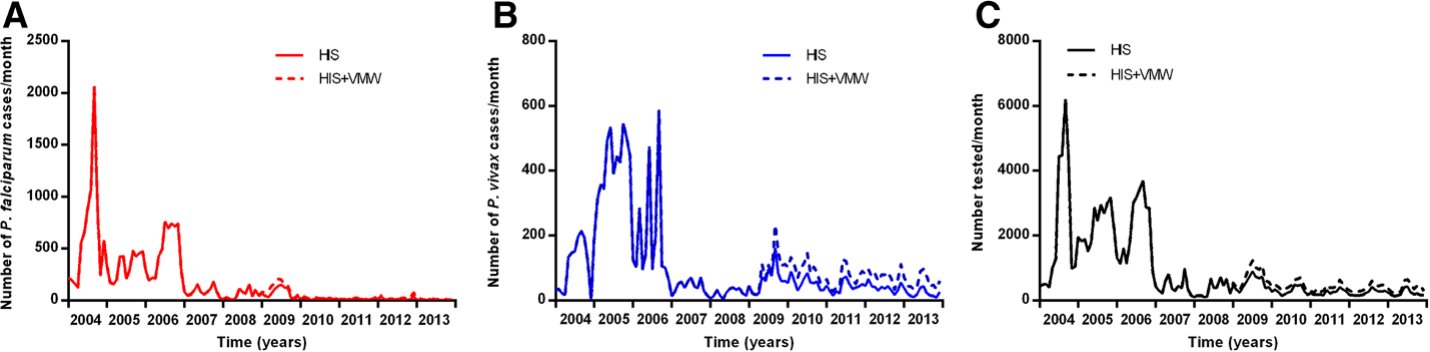
**Additional monthly malaria cases detected by VMWs in Pailin by species. A** *P. falciparum* and **B** *P. vivax* and **C** number tested for malaria and % of tests positive.

There have been VMWs in Pailin Province since 2009. They have gradually increased in number to cover all 114 villages with 228 VMWs and an additional 45 mobile malaria workers covering 30 of the 114 villages by the end of 2013. In addition, long-lasting insecticide-treated bed nets were distributed in 2009 in Pailin Province, with virtually 100% coverage of households by the end of that year. The number of *P. falciparum* cases detected by VWMs in Pailin Province greatly decreased from 2009 onwards, paralleling the decrease seen in the HIS data (Figure 14A). The number of people with *P. vivax* and number of people tested for malaria did not change from 2009 to 2013 (Figures 14B and C).

**Figure 15:**
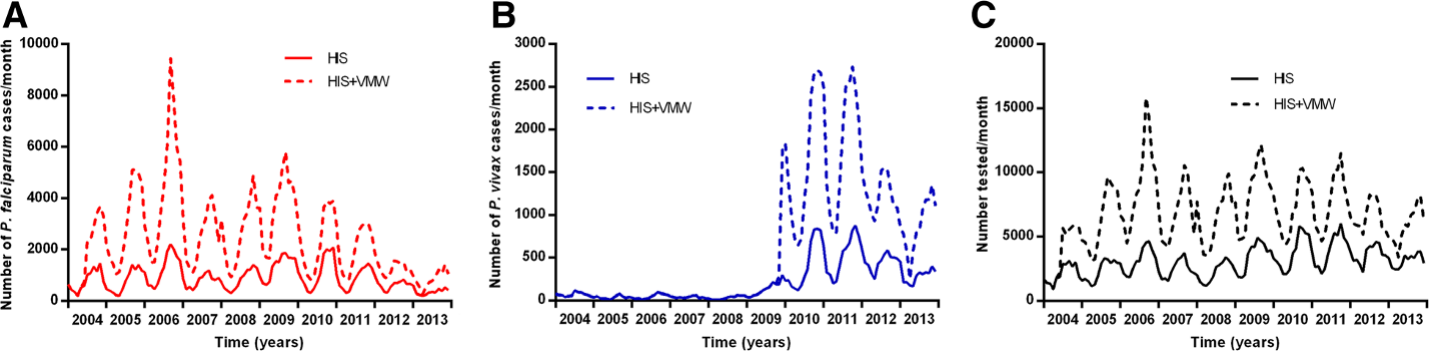
**Additional monthly malaria cases detected by VMWs in the northeast of Cambodia by species. A** *P. falciparum* and **B** *P. vivax* and **C** numbers of positive tests and % of tests positive.

In the northeast of Cambodia, VMWs started work in 2004 following small-scale pilots and the number of cases of *P. falciparum* detected by them remained steady until 2009 (Figure 15A). Numbers of *P. vivax* detected by VMWs in the Northeast increased from mid-2009 (Figure 15B). The number of people tested each year did not change (Figure 15C). These patterns seen in the MIS paralleled those reported through HIS.

## Discussion

The overall number of cases of *P. falciparum* and *P. vivax* malaria attending public sector facilities in Cambodia has fallen markedly by 59% since 2011. This coincided with a rapid scale-up of distribution of insecticide treated bed nets and coverage of villages in high-risk areas by village malaria workers. *P. falciparum* remained relatively stable from 2004–2011 whilst numbers of *P. vivax* cases increased despite substantial distribution of ACT and the roll-out of RDTs during that period. The reasons for this delayed fall in cases are unclear but further examination is warranted to derive lessons for malaria control programmes elsewhere. The fall in percent of tests for malaria which were positive since 2011 with similar annual numbers tested throughout suggests the decline in incidence of clinical malaria was real and not an artefact due to changing surveillance efforts. Additionally, it appears that more effort has been put into testing to detect the same number of cases, particularly in the low season. This may be masking an earlier actual decline in *P. falciparum* in 2010 and 2011.

The marked increase in *P. vivax* in this study began a year too early for it to be related to the decrease in *P. falciparum*[11, 12]. Alternatively, it could be a result of improved diagnosis with introduction of a HRP-2/pLDH RDT (CareStart™) in 2009 in place of a Pf only HRP2 RDT (Paracheck Pf®), improved microscopic differentiation of species on diagnosis and better sensitivity of testing with development of the malaria control programme and improvements in training of microscopists from 2009 [13]. Other differences between *P. falciparum* and *P. vivax* included different timing of seasonal peaks in cases and differences in the geographical distribution in numbers of cases over time. A greater relative decline in cases of *P. falciparum* than *P. vivax* and differences in the periodicity were also seen in Peru, particularly in mountain and jungle regions with some influence of precipitation and temperature [14]. In Cambodia, further investigation of the influence of climatic factors and malaria control measures on the two species over space and time would be informative.

The decrease in mortality found in this study is likely to be due to improvement in healthcare provision during the study period. The difference in mortality in VMW and non-VMW areas in the HIS data suggest that VMWs may have contributed to this decreased mortality, presumably through earlier effective diagnosis, treatment and onward referral at a village level.

The roll-out of VMWs across remote and high transmission areas of Cambodia has resulted in detection of a large previously unrecorded burden of disease. With the scale-up of VMWs to over 40% of the villages in the higher transmission areas of Cambodia in the past five years, the total number of detected cases of both species nationally has almost doubled. Numbers of people reported through HIS did not fall during the roll-out of VWMs. This suggests that many of those people now presenting to VMWs previously sought care in the private sector and that the cohort who previously accessed public sector healthcare services for diagnosis and treatment of malaria continue to do so. If VMWs were rolled out further, additional increases in the detected caseload would thus be expected. VMWs have the advantage of being accessible to villagers who may not otherwise attend public sector facilities with their malaria. In the absence of VMWs, a high proportion of individuals are thought to purchase anti-malarials from the private sector. This was estimated at 87% in a survey in 2002 [15] and 54% in 2011 [16]. Despite the intensified malaria control efforts in and around Pailin, 43% were estimated to have received treatment from the private sector in 2011 [16]. It is likely that many of these will choose instead the free testing and treatment available from the VMWs as had been shown previously [15] and in a recent evaluation by CNM [17]. As data were not available on attendees at private pharmacies and clinics during the time of the study, the total burden of malaria in Cambodia remains unclear although it is evident from these data that it is grossly underestimated. This was suggested in a previous study, which showed that this underestimate is most prominent in remote areas of the country [18]. A conservative estimate for total malaria burden in Cambodia from the present study would be over 100,000 cases per year from 2004–2011, falling to 70,000 in 2012 and 42,000 in 2013. These figures do not include those who sought treatment in the private sector (conservative estimates being over 50%) and it is not clear how many of these would attend a VMW if available. In 2013, VMWs were present in 44% of the villages in ODs with high malaria prevalence. If malaria prevalence across all villages in these ODs with VMWs were similar, then the actual number of cases could be 1/0.44 = 2.3 times the current number detected by VWMs i.e. 47,000. This would give a total of around 68,000 malaria cases per year in Cambodia in 2013, an API of 4.6 per 1000. Similarly for 2012, the estimated API using this method would have been 7.5 per 1000. This is 2.8 times higher than the estimate from WHO for that year of 2.7/1000 [19]. In 2015, the private sector will no longer be permitted to diagnose or treat malaria. A more accurate picture of malaria burden in Cambodia can be expected as a result. By providing free testing and treatment, VMWs could be expected to increase the number of people being treated for malaria. This should lead to a decrease in transmission overall in areas with VMWs. This study confirmed this with a greater decrease in cases of *P. falciparum* in ODs with VMWs compared to ODs with no VMWs. Malaria mortality was also lower in ODs with VMWs highlighting the benefits of early effective diagnosis and treatment (EDAT).

During the study period, the geographical distribution of malaria in Cambodia has changed. This is likely to be due to a combination of factors including changes in malaria control activities, migration and deforestation. In some areas, particularly in parts of the west of Cambodia, *P. falciparum* decreased. Pailin Province had the greatest decrease in *P. falciparum* cases from 2007 onwards. It is not clear which factors caused this decrease although it coincided with the beginning of a period of intense focus on Pailin as the location of newly discovered artemisinin resistance [20]. This resulted in intensification of malaria control activities in the area including increased availability of ACT and intensified active surveillance, such as focused and mass screening and treatment (FSAT and MSAT). It was also around this time that long-lasting insecticide treated bed nets were introduced [21]. Additionally, extensive deforestation has occurred in Pailin during this period, resulting in very little forest cover in 2013. Before 2007, Pailin was the OD with the highest malaria transmission in Cambodia (API/1000 = 244 in 2004). That the surrounding Province of Battambang experienced a coincident decline in malaria, whilst other parts of Cambodia did not, may reflect the decreasing influence of the Pailin malaria ‘hot spot’ on adjacent areas. As of 2012, every village in Pailin has a VMW and this is likely to result in both improved collection of malaria data and a further decrease in the burden of disease through EDAT and directly-observed therapy (DOT). It is perhaps reassuring that the large decrease in malaria burden in Pailin was achieved despite the presence of reduced parasite clearance rates with artemisinins. However, reductions in disease burden were much less in Pursat Province where artemisinin resistance has also been identified [22] and bed net coverage has been high. It is possible that this difference between Pursat and Pailin is due to there having been far fewer VMWs and less deforestation in Pursat.

The northeast of Cambodia differed from the rest of the country with an increase in *P. falciparum* from 2007–2010 and, despite recent decreases in disease burden, is now the area with highest transmission intensity. There was also an even larger proportional increase in *P. vivax* than that seen nationally since 2009. Reasons for this are not clear. The Northeast is a relatively remote area with remaining high forest cover and some parts have had relatively lower levels of malaria control activity than elsewhere in Cambodia. Although VMWs have been present in the Northeast since 2003, there have been relatively few in 2 of the 4 provinces (Steung Treng and Kratie). Additionally few ITNs had been distributed in Steung Treng to the end of 2012, although the rest of the Northeast had a high proportion of households with ITN. The main focus for malaria control recently has been on Pailin and other areas with or suspected to have artemisinin resistance [23]. Changing seasonal migration between the Northeast and the highly populated south of the country is one possible factor worth exploring further [24]. The contribution of ongoing deforestation in parts of Cambodia to the reduction in malaria also needs to be investigated further.

In 2013, the Provinces bordering Laos, Vietnam and Thailand in the north and northeast of the country had the highest transmission intensity for both species. These borders are frequented by migrant and mobile populations who have proven difficult to target for surveillance and malaria control activities. A recent study by CNM found 14% of individuals crossing the Cambodia-Laos border at one location in Steung Treng to be positive for malaria by polymerase chain reaction (PCR) and two thirds of these were asymptomatic (unpublished data). This raises the possibility that importation of malaria from neighbouring countries is contributing to the higher incidence of infection found in the border areas of Cambodia. Migrant populations more often delay seeking appropriate diagnosis and treatment and are a potential source and means of dispersal of anti-malarial drug resistance, which is frequently first identified in border areas [25, 26].

This study had several limitations. This study did not measure transmission intensity directly. Instead, API was used as a surrogate measure. Although the majority of cases are likely to have been infected in the same district as where they were diagnosed, it should be noted that for some individuals transmission may have occurred elsewhere. Malaria surveillance data are notoriously prone to collection bias. Self-treated and privately treated cases including those treated by private pharmacies and traditional healers were not captured by these data and migrant and mobile populations were probably underrepresented. Although it is likely that many of these underrepresented cases chose to attend VMWs where available, the true numbers of such cases are not known. Remote populations are also thought to have been underrepresented by the HIS data [18]; however, most of these communities are now included in the VMW project. Changes in efficiency of the systems used to collect the data are difficult to quantify and may introduce biases. The strength of these data is that several trends are apparent over time in the two independent datasets. During the study period, there was widespread deforestation, and the contribution of this to the reduction in malaria, transmitted predominantly by forest-dwelling mosquitoes was not examined in this study.

This analysis highlights a number of areas requiring further detailed study. Further investigation of trends over time in individual ODs matched with details of malaria control interventions employed, changes in environmental determinants and investigation of the contribution of mobile and migrant populations would be highly informative for planning future malaria control activities. In particular detailed data on ACT use have been collected in large national surveys and collection of information on the behavior and malaria burden in mobile and migrant populations (MMPs) is ongoing and a National MMP Strategy has been developed [27]. The VMW project collects more detailed data than we have had space to present here in this paper including age, gender, migration status, pregnancy status and breakdown by individual village. This growing dataset is an invaluable resource for the future for Cambodia. By combining these data with other studies currently being undertaken, for example, on population migration and the geographical distribution of artemisinin resistance, important new insights will be gained.

## Conclusion

This study has identified several major trends in malaria caseload in Cambodia over the past decade which warrant further investigation. This includes a marked decrease in *P. falciparum* malaria since 2009 coincident with scale-up of insecticide treated bed nets and village malaria workers. Although malaria surveillance data are notoriously prone to collection bias, the use of data from two independent sources greatly increases the robustness of the findings.

## Electronic supplementary material

Additional file 1: Video 1: Spatial distribution of *P. falciparum* malaria by OD in Cambodia from 2004 to 2013. API per 1000 population. (AVI 52 MB)

Additional file 2: Video 2: Spatial distribution of *P. vivax* malaria by OD in Cambodia from 2004 to 2013. API per 1000 population. (AVI 52 MB)

## Abbreviations

ACT: Artemisinin combination therapy
API: Annual parasite incidence
CNM: National Centre for Parasitology, Entomology and Malaria Control
DOT: Directly observed therapy
EDAT: Early effective diagnosis and treatment
HIS: Health Information System
ITN: Insecticide-treated bed net
LDH: Lactate dehydrogenase
MIS: Malaria Information System
MMP: Mobile and migrant populations
MMW: Mobile malaria worker
OD: Operational district
PCR: Polymerase chain reaction
Pf: *P*. *falciparum*
p-HRP2: Parasite histidine-rich protein 2
Pv: *P*. *vivax*
RDT: Rapid diagnostic test
VMW: Village malaria worker.
